# Composting Dynamics, Bedding Properties, and Seasonal Effects in Composting and Non-Composting Bedded-Pack Barns in a Subtropical Region

**DOI:** 10.3390/ani16111745

**Published:** 2026-06-05

**Authors:** Beatriz Danieli, Maksuel Gatto de Vitt, Fábio José Gomes Bertipaglia, Juliano Vitória Domingues, Aline Zampar, Maria Luísa Appendino Nunes Zotti, Patrícia Ferreira Ponciano Ferraz, Ana Luiza Bachmann Schogor

**Affiliations:** 1 Graduate Program in Animal Science, Santa Catarina State University (UDESC), Chapecó 89815-630, Brazil; beatriz.danieli@udesc.br (B.D.); mak-witt@hotmail.com (M.G.d.V.); aline.zampar@udesc.br (A.Z.); maria.anunes@udesc.br (M.L.A.N.Z.); 2 Department of Veterinary Medicine, University of West Santa Catarina, Campos Novos 89620-000, Brazil; gomes.fj@outlook.com; 3 Instituto do Meio Ambiente de Santa Catarina (IMA/SC), Chapecó 89802-120, Brazil; juliano.tche@gmail.com; 4 Department of Agricultural Engineering, Federal University of Lavras (UFLA), Lavras 37200-000, Brazil; patricia.ponciano@ufla.br

**Keywords:** bedding temperature, composting dynamics, dairy cows, seasonal climate variation

## Abstract

This study investigated the effects of construction design and seasonal climatic conditions on bedding dynamics in bedded-pack dairy systems with contrasting composting functionality. The study intentionally included systems representing both composting bedded-pack barns (CBP), characterized by active management (regular turning and ventilation), and non-composting bedded-pack barns (BPB), which lacked aeration and did not promote active composting, resulting in limited or absent composting activity. Nine farms were divided into three groups: CONV (large, full-time CBP), ADAP (adapted, full-time CBP), and PART (partially used BPB). Evaluations were conducted during both cold and hot seasons. Composting dynamics were assessed over 24 h by measuring bedding temperature and moisture at eight points. During daytime, additional measurements at twenty points allowed for spatial distribution analysis using the inverse distance weighting method. Bedding attributes—including pH, density, depth, and particle size—were also measured in eight points. A 2 × 3 factorial design (two seasons, three barn types) was applied, and data were analyzed using Tukey’s test and Pearson correlation. Microclimate conditions were monitored through air temperature and humidity. Bedding temperature was significantly higher in the hot season (36.55 °C) compared to the cold season (32.12 °C), and was highest in the ADAP group (40.01 °C), followed by CONV (37.39 °C) and PART (26.18 °C) (*p* < 0.05). The 24 h temperature curve indicated favorable composting conditions only in the CONV and ADAP groups. Spatial temperature distribution varied significantly across locations in most barns (*p* < 0.05). Moisture content was lower in the hot season (46.91% and 41.41%) than in the cold season (57.03% and 51.97%) for CONV and ADAP, respectively. Moisture and temperature were significantly correlated with key bedding characteristics (*p* ≤ 0.05). Overall, a greater combination of characteristics associated with more favorable composting conditions was observed in ADAP barns, particularly during the hot season, whereas PART systems showed conditions incompatible with active composting.

## Simple Summary

This study evaluated how barn design and seasonal conditions influence bedding dynamics in bedded-pack dairy systems with contrasting composting functionality. Nine farms were classified into three barn types: CONV (large conventional compost bedded-pack barns), ADAP (adapted compost bedded-pack barns), and PART (partially used bedded-pack barns without active composting management). Evaluations were conducted during the cold and hot seasons, measuring bedding temperature, moisture, and physical characteristics. Bedding temperature was higher in the hot season and in CONV and ADAP barns, indicating more favorable composting conditions in these systems. In contrast, PART barns consistently showed lower temperatures and higher moisture, suggesting limited or absent composting activity. Moisture was lower during the hot season, and significant relationships were observed between temperature, moisture and bedding characteristics. Overall, composting performance in compost bedded-pack barns (CBP) under humid subtropical conditions was strongly influenced by the interaction between barn design, ventilation, and bedding management. Systems with regular turning and mechanical ventilation maintained more stable thermal and moisture conditions, whereas non-composting bedded-pack systems showed limited or absent composting activity. These findings highlight the importance of integrating structural design and management practices to ensure effective bedding function in subtropical dairy systems.

## 1. Introduction

Bedded-pack barns (BPB), including both composting and non-composting configurations, are considered effective housing systems for mitigating the effects of climatic conditions on loose-housed dairy cows [1,2]. These systems provide benefits such as enhanced animal movement [3,4], manure concentration [5], and increased milk production [3].

The traditional bedded-pack barn layout, consisting of a resting area and a feeding alley [6], can be adapted to meet farm-specific logistical, climatic, and economic requirements [7]. However, successful adaptation requires management strategies that enhance bedding moisture removal, considering factors such as ventilation capacity, air exchange rate, roof height and ridge opening, solar exposure, site slope and drainage, and protection against rainfall intrusion [8]. Together, these factors govern evaporative drying potential and heat dissipation within the bedded pack and are essential for maintaining proper composting conditions in CBP systems [8]. According to Janni et al. [6], adequate ventilation (air exchange) is necessary for this kind of barn to remove the heat and moisture generated by the biologically active pack. Thus, the use of bedding material with high moisture content is not recommended, as additional energy is required for drying; moreover, bedding materials must be turned frequently [9].

In composting bedded-pack barns (CBP), the resting area functions as a biologically active composting system in which frequent aeration and appropriate moisture content create conditions that favor processes such as organic matter decomposition, oxygen incorporation, pathogen reduction, and material stabilization [10,11]. Bedding moisture is influenced not only by aeration but also by barn occupancy rate, since higher stocking density increases excreta and urine inputs and elevates moisture, whereas lower density favors drying [8]. Composting efficiency thus reflects the balance between moisture inputs and evaporative losses governed by ventilation effectiveness, air renewal, and barn geometry [9].

In systems with active composting (CBP), bedding temperature and moisture are key indicators of microbial activity: temperature at depth reflects decomposition intensity [6,12], whereas moisture regulates oxygen availability and microbial metabolism [12]. Inadequate control of these interacting factors frequently results in excessive moisture and suboptimal composting.

Design and management guidelines for composting bedded-pack barns have been proposed in the United States, Europe, and Brazil [4,8,13,14,15,16,17], yet their effectiveness depends on regional climate. In Brazil, the humid subtropical climate (Cfa), characterized by high rainfall, elevated humidity, and warm summers, predominates in the southern region [18]. These conditions reduce evaporative drying, limit natural ventilation efficiency, and increase the risk of rainwater ingress and persistent bedding moisture, thereby challenging bedded-pack management and animal heat dissipation [4,7,16].

National assessments indicate that bedded-pack barns can improve technical and economic performance when properly implemented, but outcomes remain highly dependent on barn design and seasonal climatic variability [19]. Radavelli et al. [16] surveyed several bedded-pack systems in southern Brazil and were able to classify them into three groups with different levels of composting management intensity, based on the influence of size, use frequency, and barn structural characteristics on bedding properties evaluated at a given time. However, interactions between barn structural design and seasonal climate on composting dynamics remain poorly documented, particularly under humid subtropical conditions. Furthermore, the extent to which the climate of this region influences the spatial and temporal variability of the bedding during cold and hot seasons is still unknown.

We therefore hypothesize that, in subtropical regions, barn design and seasonal climate jointly modulate composting dynamics and barn microclimate. Accordingly, the objective of this study was to evaluate how seasonal climatic variability influences the spatial and temporal dynamics of bedding in bedded-pack systems with contrasting composting functionality (composting vs. non-composting), thereby allowing the comparison between systems with active composting (CBP) and systems without composting (BPB). Additionally, this study aimed to provide practical insights into bedding management and barn configuration that improve moisture control, aeration, and overall composting performance in CBP systems.

## 2. Materials and Methods

### 2.1. Farms

This study was conducted during the cold season (August to October 2018) and the hot season (January to March 2019) on nine dairy farms with bedded-pack barn systems in Santa Catarina State, Brazil. The study intentionally included systems with contrasting levels of composting management intensity. The classification of bedded-pack barns into CONV (large conventional), ADAP (adapted), and PART (partially used bedded pack) groups was based on a previous multivariate cluster analysis that integrated structural characteristics, management practices, and stocking-related variables [16]. Thus, the evaluated systems represent functionally distinct bedded-pack typologies with different composting functionality (CBP vs. BPB), rather than isolated effects of barn design or management factors.

Farm selection and farmer participation followed a voluntary and collaborative approach. Farmers were initially contacted through cooperative partnerships, and preliminary telephone conversations were conducted to explain the objectives and procedures of the study. Eligible farms were identified based on structural characteristics previously described by Radavelli et al. [16], and all selected farms agreed to participate.

All participating farmers provided informed consent for farm visits, data collection, and the use of information for scientific publication. The study procedures complied with ethical and collaborative requirements, ensuring confidentiality and transparency throughout the research process. Each farm was visited for four consecutive days in each season.

In this study, bedded-pack barns were defined according to Radavelli et al. [16], encompassing systems with an organic bedding pack that may operate either with active composting management (CBP) or without composting processes (BPB) and not merely barns with a bedded resting area and feeding alley. The study categorized bedded-pack barns in Brazil into three groups with similar characteristics, which informed the selection of the nine farms for this study. Each group adhered to the classifications established by Radavelli et al. [16]:

(a) The CONV group consisted of large conventional compost bedded-pack barns (CBP) (Farms 1–3) that were permanently used, exclusively featuring new and larger barns (bedded-pack areas ranging from 1301 to 2800 m^2^), with regular bedded-pack turning and mechanical ventilation, and similar to the American models outlined by Janni et al. [6].
(b) The ADAP group included conventional or adapted compost bedded-pack barns (CBP) that were also permanently used, with regular bedded-pack turning and mechanical ventilation. These barns could be constructed according to the layout defined by Janni et al. [6], provided that they had bedded-pack areas between 285 and 1300 m^2^ or were adapted from other rural facilities with varying bedded pack sizes.
(c) The PART group comprised non-composting bedded-pack barns (BPB), characterized by intermittent occupation, access to grazing areas, and absence of mechanical ventilation and bedding turning. Regardless of size, these barns were utilized only during the hottest parts of the day or during the rainy season, lacking mechanical ventilation. Although previously classified as bedded-pack systems [16], in the present study these barns were not considered composting systems due to the absence of aeration and frequent bedding turning, resulting in limited or absent composting activity.

The CONV group’s stables (Farms 1–3) in this study had bedding areas ranging from 900 to 1500 m^2^. All evaluated farms consisted of open front barns, had precast concrete structures and metal roofs. Farms 1 and 3 had low walls on the outside perimeter to contain the bedding (approximately 90 cm high), whereas this feature was absent in Farm 2. Mechanical ventilation was present in all facilities, installed either in the holding area or along the feed alley; sprinklers were available only in Farm 2. Farms 1 and 2 used twice-daily milking, whereas Farm 3 used an automatic milking system. Across the manuscript, this group is referred to as “large, fully confined bedded-pack barns with continuous use and standardized infrastructure”.

The ADAP group’s stables (Farms 4–6) had bedding areas ranging from 975 to 1470 m^2^. All evaluated farms consisted of open front barns. Farm 5 followed the dairy layout of Janni et al. [6], whereas Farms 4 and 6 were converted from broiler barns and lacked standardized design. Farm 5 had low walls on the outer perimeter to contain the bedding (approximately 90 cm high), whereas this feature was absent in Farms 4 and 6. Mechanical ventilation was present in all barns, but sprinklers in the feeding lane occurred only in Farm 4. Farm 5 milked twice daily, whereas Farms 4 and 6 milked three times daily. This group is described as “smaller or adapted bedded-pack barns in continuous use, with variable construction standards”.

The PART group’s stables (Farms 7–9) had bedding areas ranging from 220 to 480 m^2^. Provided cows with access to grazing areas, and no active compost management. All evaluated farms consisted of open front barns. Farms 7–9 used bedded-pack barns intermittently, with cows having daily access to pasture. Farm 7 had low walls on the outer perimeter to contain the compost (approximately 90 cm high), whereas this feature was absent in Farms 8 and 9. Barns were mainly occupied during hot periods or rainfall and had no mechanical ventilation or bedding turning. Milking occurred twice daily. Grazing access ranged from 14 to 22 h d^−1^ (Farms 7–9: 20, 14, and 17 h, respectively). Farms 7 and 8 used the barn primarily for supplementation with corn silage and concentrate, in addition to providing shelter during hot periods or rainfall, whereas Farm 9 allowed voluntary sheltering during the hottest hours to provide shade. This group is referred to as “partially used non-composting bedded-pack barns (BPB) combined with grazing, without ventilation or bedding management”.

**Figure 1:**
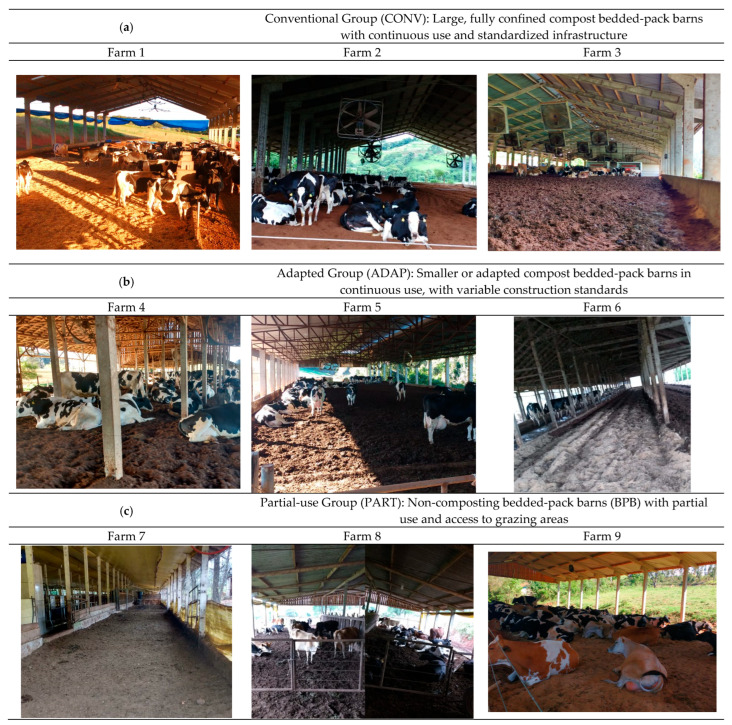
Representative images of the bedded-pack barn systems across the nine farms, illustrating structural characteristics and bedding conditions within the (**a**) CONV and (**b**) ADAP compost bedded-pack barns, and (**c**) PART (BPB) groups.

The characteristics of the farms, stables, the bedding turning and bedding material of each farm are presented in Table 1. Additional characteristics of the nine bedded-pack systems can be observed in Figure 1.

### 2.2. Bedding Characteristics

**Figure 2:**
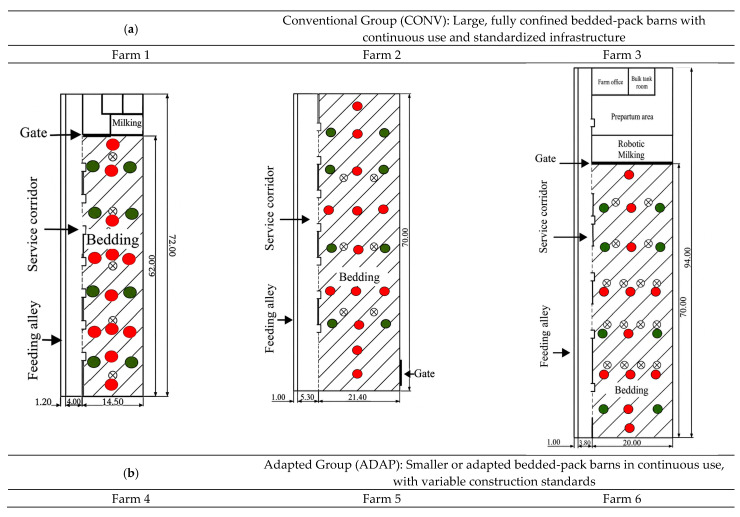

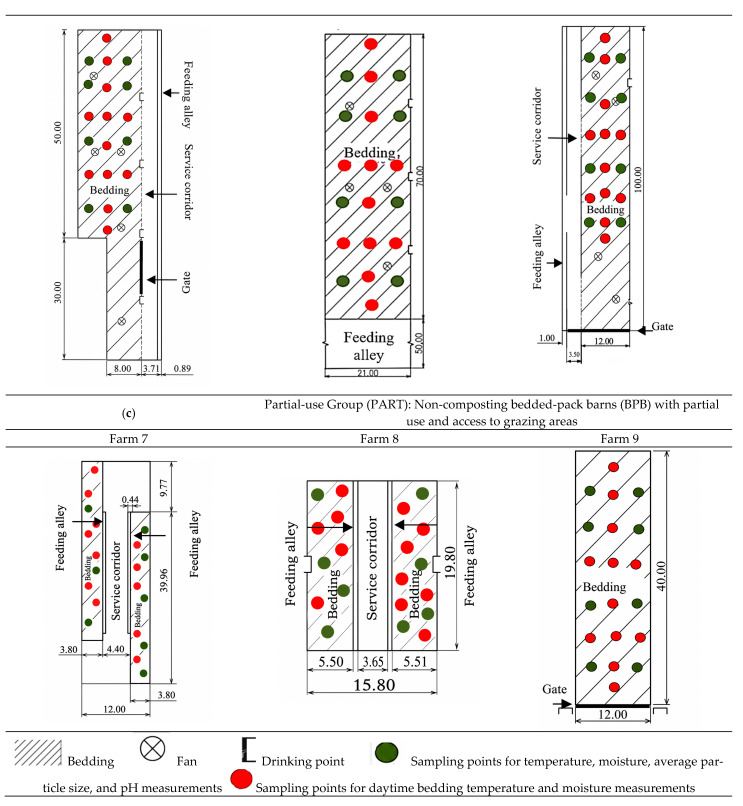
Site maps showing the location of the sampled points in the nine bedded-pack barns belonging to the (**a**) CONV and (**b**) ADAP compost bedded-pack barns, and (**c**) PART (BPB) groups.

Sampling locations within each bedded-pack are shown in Figure 2. Fixed internal reference markers were established within each bedded-pack to guide point positioning and ensure that repeated measurements were consistently collected at the same locations throughout the study. Eight equidistant points (green circles) were used to evaluate bedding characteristics over four consecutive days. Temperature was measured in situ at 0.2 m depth (with the exception of the CBP on Farm 9, where the depth was insufficient; therefore, measurements were taken at a depth of 0.10 m), whereas samples for moisture, average particle size (APS), and pH were collected at the same depth immediately before and after bedding turning. The evaluation at 0.20 m depth was chosen because this layer represents the most biologically active zone of the bedding, where microbial activity and composting processes are more stable and less influenced by short-term surface fluctuations (e.g., ambient temperature and recent manure deposition) [17]. Each sample was analyzed individually in the laboratory, resulting in two observations per point and sampling time (pre- and post-turning). For statistical analysis, the mean of these paired measurements was used.

Bedding depth and bulk density (kg m^−3^) were measured once at each of the eight primary sampling points before turning. Bedding depth was determined using a graduated metal rod. Bulk density was measured using a cylindrical probe (12 cm diameter × 30 cm length) inserted to a depth of 0.20 m to collect a known volume of bedding material. Sampling was performed under in situ conditions, without additional compaction or leveling of the material. The collected sample was placed in a pre-weighed metal container, weighed to obtain wet mass, and bulk density was calculated as the ratio of mass to the sampled volume, following ASAE Standard S269.4 DEC 91 [20].

The combined weight of the material and the container was recorded, and the bulk density was calculated using Equation (1):

(1) $$Bulk\;density\;(kg\;m^{-3})=\frac{M}{V}$$

where *M* is the mass of the tested bedding material (kg) and *V* is the container volume (m^3^).

Temperature was recorded continuously for 24 h in PART farms. In CONV and ADAP farms, data loggers were removed during bedding turning (≈15 min), and these intervals were excluded from analysis.

An additional twelve points (red circles), combined with the eight primary points, totaled twenty locations for daytime assessment of bedding temperature and moisture at 0.2 m depth over two consecutive days (Figure 2). Moisture samples were likewise collected at 0.2 m depth immediately before and after bedding turning and analyzed following the same laboratory procedures. However, unlike the other bedding variables, moisture and temperature data were retained as separate pre- and post-turning observations to evaluate changes in bedding moisture and temperature associated with turning events.

Temperature was logged every 10 min using DS1922T iButton® temperature data logger (Maxim Integrated Products, Inc., San Jose, CA, USA; resolution 0.0625 °C; range −40 to 85 °C). Sensors were protected with a latex covering to prevent direct contact with moisture content. Immediately after bedding turning, the predefined sampling location was identified, and the insertion depth (0.20 m) was measured using a graduated ruler (with the exception of the bedded-pack on Farm 9, where the depth was insufficient; therefore, measurements were taken at a depth of 0.10 m). A small opening was made in the bedding, and the sensor was placed at the depth, attached to a colored strip to facilitate later identification and retrieval. The sensor was then covered with bedding material and remained in place until the next turning event. Data were downloaded using OneWireViewer^®^ (Maxim Integrated Products, Inc., San Jose, CA, USA).

Samples for pH, moisture, and APS were stored in insulated containers at 5 °C until laboratory analysis. Methods followed Silva and Queiroz [21] for pH and moisture, and Damasceno [22] for APS.

Bedding material was allowed to air-dry for 48 h prior to particle size distribution analysis. The dried compost was sieved using an electromagnetic sieve shaker (Bertel Indústria Metalúrgica^®^, Caieiras, SP, Brazil). The material was placed in graduated volume cylinders and processed through a series of vertically aligned sieves with decreasing mesh openings (9.50 mm, 4.75 mm, and 2.00 mm), with a pan at the bottom. The bedding material was sieved for 3 min. The material retained on each sieve was transferred to a beaker and weighed. The total compost bedding sample weight (*W_total_*) was calculated as the sum of the weights retained on all sieves, including the bottom pan [23]. So, *W_total_* is given by Equation (2):

(2) $$W_{total}(g)=\;W_{coarse}+\;W_{4.75}+\;W_{2.00}+\;W_{fines}$$

where *W_coarse_*, *W*_4.75_, *W*_2.00_, and *W_fines_* represent weights ≥ 9.50 mm, <9.50 mm ≥ 4.75 mm, <4.75 mm ≥ 2.00 mm, and <2.00 mm, respectively.

APS fractions for ranges (≥9.50; <9.50 ≥ 4.75; <4.75 ≥ 2.00; and <2.00) were determined by Equation (3):

(3) $${APS}_{fractions}\left(\%\right)=\;\frac{W_{\left(X\right)}}{W_{total}}\times \;100$$

where *W_(x)_* represents the weight for particles sizes of the four ranges (≥9.50; <9.50 ≥ 4.75; <4.75 ≥ 2.00; and <2.00).

### 2.3. Microclimatic Characteristics

The thermal environment was continuously monitored by recording dry-bulb air temperature (tdb, °C) and relative humidity (RH, %) at 15 min intervals throughout the experimental period. Inside each barn, HOBO U12-013 sensors (Onset Computer Corp., Bourne, MA, USA) were installed 2 m above the bedding surface at the geometric center of the structure to represent internal conditions while avoiding interference from machinery movement. External climate was measured using the same sensor model placed 1.5 m above ground within a standard meteorological shelter located 10 m from the barn. Sensors had a temperature range of −20 to 70 °C (±0.35 °C) and RH range of 5–95% (±2.5%).

### 2.4. Floor Plans and Spatial Distribution Mapping (Bedding Temperature and Moisture)

Bedded-pack barn layouts were drawn to scale in AutoCAD 2016 [24], including structural dimensions and georeferenced sampling locations (Figure 2). The spatial distribution of bedding temperature and moisture, obtained from 20 sampling points, was analyzed using inverse distance weighting (IDW) interpolation. This deterministic method estimates values at unsampled locations based on the assumption of spatial autocorrelation, whereby observations closer in space are more similar than those farther apart. Thus, surrounding sampled points exert distance-decaying influence on prediction locations, generating continuous isovalue surfaces that represent spatial variability across the bedding area. As an exact interpolator, IDW preserves sampled values at their original locations and tends to produce localized zones of influence around data points [25]. Geostatistical spatial modeling techniques have previously been applied to evaluate distribution patterns and spatial dependence among sampling locations in similar studies [1,26]. IDW was implemented in QGIS [27] using a power parameter (*p* = 2), selected through cross-validation as the most suitable for local conditions. Interpolated raster was produced with a spatial resolution of 2 m per pixel. For comparative analysis between winter and summer, temperature and moisture surfaces were classified into six equal-interval classes, considering the observed ranges of 20–50 °C and 14–68%, respectively.

### 2.5. Statistical Analysis

Data were analyzed using a randomized block design organized in a 2 × 3 factorial scheme, considering two climatic seasons (hot and cold) and three groups (CONV, ADAP, and PART). To account for the inherent variability in facility infrastructure and management, the three bedded-pack barn system types were defined as blocks: (a) CONV, (b) ADAP, and (c) PART. This blocking strategy was adopted to isolate the “design effect” (barn architecture, ventilation, and tilling dynamics) from the experimental error. Repeated measurements collected over time and across sampling points were averaged prior to analysis in order to obtain representative values for each experimental unit and to meet the assumptions of independence required for ANOVA. Prior to analysis, the normality of residuals and homogeneity of variances were assessed using the Shapiro–Wilk and Levene tests, respectively. The variables moisture content and pH did not show a normal distribution (*p* < 0.05) and were therefore log-transformed (log_10_(x)) to meet parametric assumptions. All other bedding attributes (temperature, APS, density, and depth) met the normality criteria. The effects of group, climatic season, and their interaction were evaluated via Analysis of Variance (ANOVA), and treatment means were compared using the Tukey test at a 5% significance level.

To address the spatial and temporal relationships among bedding variables, Pearson correlation coefficients (PROC CORR, SAS^®^, version 9.4, SAS Institute Inc., Cary, NC, USA) were calculated (*p* < 0.05). Linear and second-order polynomial regression models (y = β_0_ + β_1_ x + β_2_ x^2^ + ε) were fitted using R software version 3.5.0 [28] to describe the influence of air temperature and relative humidity on bedding temperature and moisture. Model selection was based on the significance of coefficients and the highest adjusted coefficient of determination (adjusted R^2^).

Hourly means of environmental and bedding variables (daily patterns) were calculated to generate daily nictemeral curves, characterizing the thermal fluctuations immediately following bedding turning events.

## 3. Results

### 3.1. Bedding Thermal and Moisture Dynamics

Mean bedding temperature differed among groups (*p* < 0.05; Table 2), with higher values observed in ADAP compared with CONV and PART. PART barns were classified as non-composting bedded-pack barns (BPB), as they lacked active compost management practices such as bedding turning and mechanical aeration. However, they were included as commercially relevant bedded-pack systems commonly observed in subtropical dairy farms in Southern Brazil. Across treatments, bedding temperatures were lower during the cold season than in the hot season (*p* < 0.05).

Moisture content showed a significant group × season interaction (*p* < 0.05; Table 2). During the hot season, moisture was highest in PART compared with CONV and ADAP (*p* < 0.05), whereas no differences among groups were observed in the cold season (*p* > 0.05). While moisture in PART remained relatively stable between seasons, CONV and ADAP exhibited higher moisture during the cold season (*p* < 0.05; Table 3).

**Figure 3:**
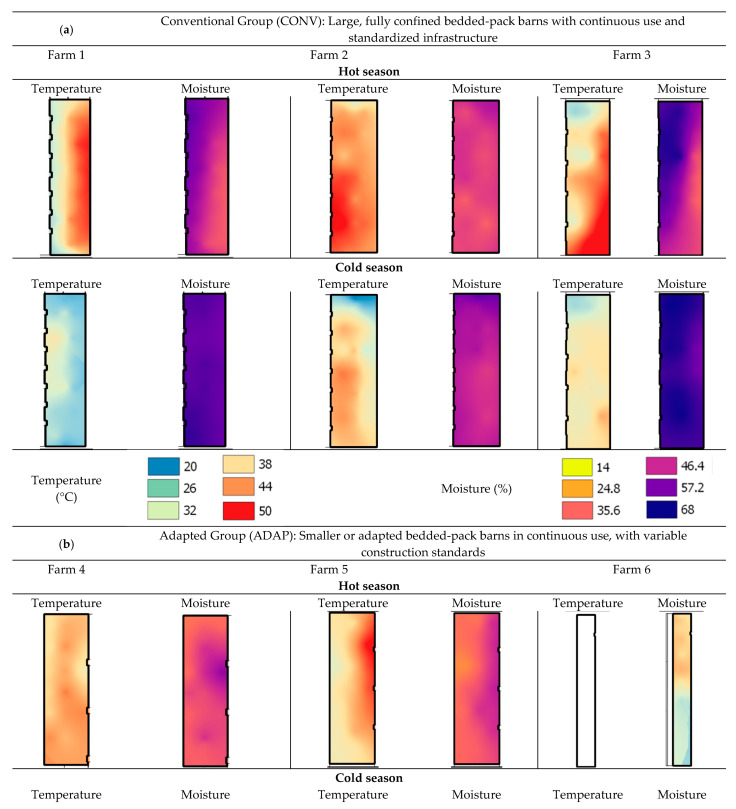

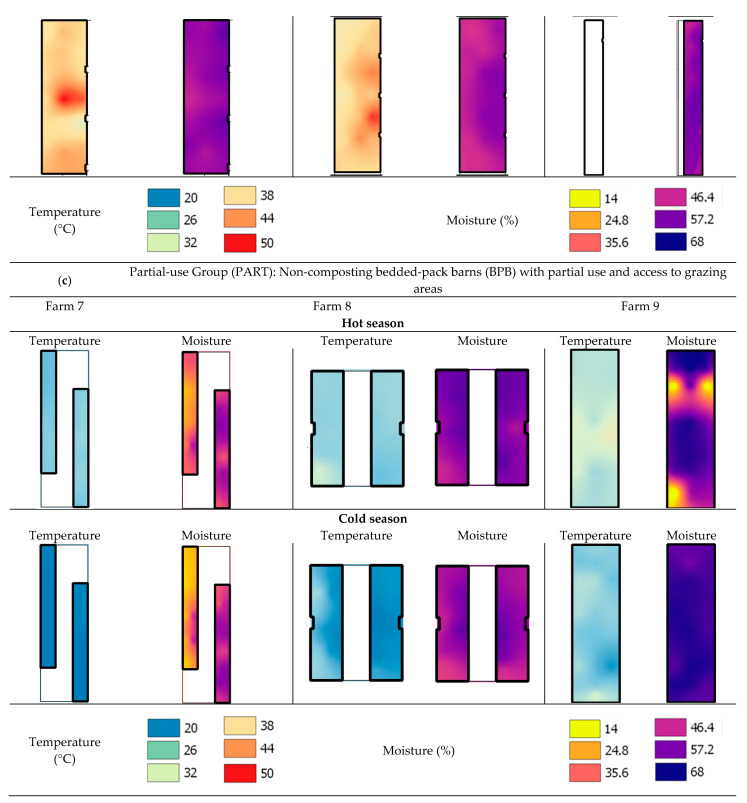
Spatial interpolation maps of daytime temperature (°C) and moisture (%) of bedded-pack barns at 0.20 m depth in Farms 1, 2, and 3 of the CONV group (**a**), Farms 4, 5 and 6 of the ADAP group (**b**) and Farms 7, 8 and 9 of the PART group (**c**) during hot and cold seasons, using IDW methods.

Spatial distributions of daytime bedding temperature and moisture are presented in Figure 3. Daytime temperatures ranged from 20 to 50 °C and were consistently lower during the cold season across all groups (*p* < 0.05). Temperature variability among sampling points was observed in most farms, except in CONV Farm 1, where differences were only detected during the hot season (*p* < 0.05). In contrast, daytime moisture showed no spatial differences among sampling points, farms, or groups within each season (*p* > 0.05). However, the group × season interaction persisted (*p* < 0.05; Table 3): during the cold season, moisture tended to be lower in ADAP and PART than in CONV, whereas in the hot season PART presented the highest moisture values, followed by CONV and ADAP, suggesting conditions incompatible with active composting in the PART group.

**Figure 4:**
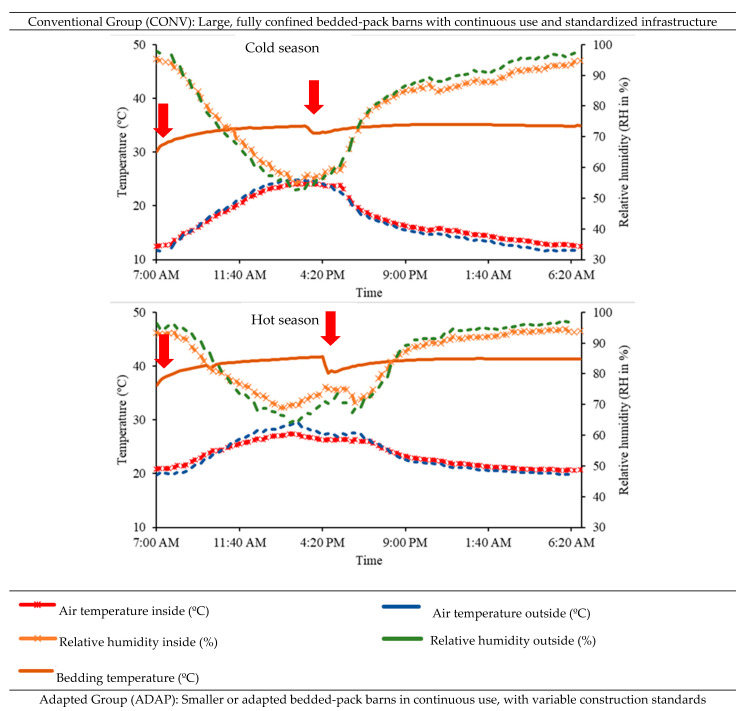

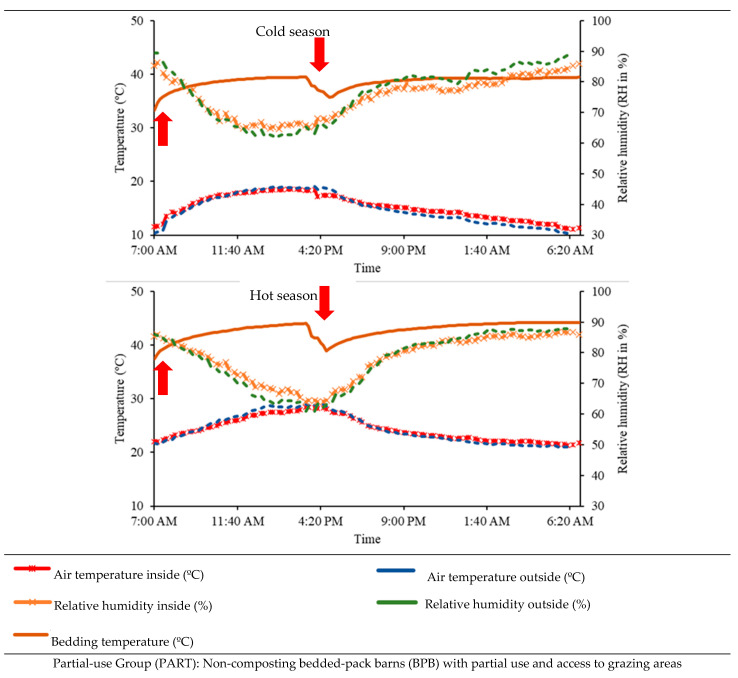

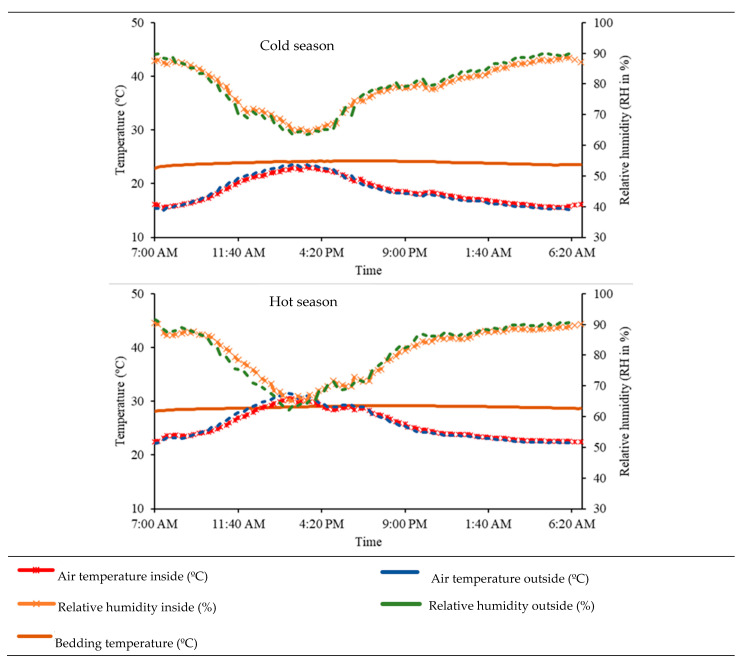
Hourly curves of average air temperature (°C), relative humidity (%), and bedded-pack temperature recorded inside and outside the barn for the CONV, ADAP, and PART groups during both hot and cold seasons. Red arrows indicate the moments when bedding turning was performed.

Daily bedding temperature curves (Figure 4) showed similar patterns in the CONV and ADAP groups, characterized by decreases immediately after bedding turning events (06:00 ± 2 h and 17:00 ± 2 h), followed by gradual increases that often reached peak values approximately eight hours later. ADAP consistently maintained higher temperatures than CONV throughout the day. In contrast, in the PART group, where bedding was not turned, temperature remained relatively stable with smaller hourly fluctuations, indicating limited or absent composting activity. In all groups, bedding temperatures were generally higher during the hot season.

### 3.2. Physical and Chemical Characteristics of the Bedding

Bedding characteristics differed among groups (Table 2). The PART group showed the highest bedding density (851 kg m^−3^) and the lowest bedding depth (0.20 m) (*p* < 0.05). In contrast, CONV (685 kg m^−3^) and ADAP (702 kg m^−3^) had similar densities, while bedding depth was greater in ADAP (0.39 m), followed by CONV (0.34 m).

Mean bedding pH also differed among treatments (*p* < 0.05), being highest in ADAP (9.75), followed by CONV (9.53) and PART (9.27). In addition, pH values were higher in the hot season compared with the cold season (*p* < 0.05).

Particle size distribution varied among groups (*p* < 0.05; Table 3). The ADAP group showed the highest proportion of intermediate particle sizes (APS ≥ 4.75 < 9.50 mm and ≥2.00 < 4.75 mm) compared to the PART group, while the CONV group did not differ from the other groups.

Significant group × season interactions were observed for proportions of coarse (APS ≥ 9.50 mm) and fine particles (APS < 2.00 mm) (*p* < 0.05; Table 3). During the cold season, no differences were observed among groups for fine particles, whereas the ADAP and PART groups exhibited higher proportions of coarse particles. In the warm season, the lowest proportion of coarse particles was observed in the ADAP group, while the PART group presented the lowest proportion of fine particles. These patterns were primarily associated with composting systems (CBP), whereas the PART group (BPB) exhibited distinct physical characteristics related to the absence of active composting.

Significant correlations were observed among several bedding attributes (Table 4). Bedding temperature was associated with moisture, pH, particle size distribution, density, and bedding depth (*p* < 0.05).

Moisture content showed positive correlations with the proportion of coarse particles (APS ≥ 9.50 mm), bedding density, and bedding depth (*p* < 0.05), and negative correlations with the proportions of intermediate (APS ≥ 2.00 < 4.75 mm) and fine particles (APS < 2.00 mm).

Weak but significant correlations were detected between pH and some particle size fractions (APS ≥ 4.75 < 9.50 mm and APS ≥ 2.00 < 4.75 mm) as well as bedding depth (*p* < 0.05). Additionally, a greater proportion of coarse particles (APS ≥ 9.50 mm) was positively associated with bedding density and depth, whereas a higher proportion of fine particles (APS < 2.00 mm) was associated with lower values for both variables (*p* < 0.05). Additional correlations among the different APS fractions are presented in Table 4.

### 3.3. Microclimatic Conditions Inside the Barns

Air temperature decreased from approximately 5:00–6:00 p.m. and remained lower until around 7:00 a.m., with similar daily patterns observed in both seasons (Figure 4). However, overall values were lower during the cold season than during the hot season. Mean air temperature was 16.78 °C in the cold season (range: 10.29–24.89 °C) and 24.50 °C in the hot season (range: 19.80–31.47 °C).

Relative humidity (RH) showed an inverse pattern to air temperature, with higher values during nighttime and early morning (00:00–07:00 a.m.; RH > 80%) and lower values during daytime hours (07:00 a.m.–5:45 p.m.; RH ≤ 70%). RH increased again from 6:00 p.m. to 11:45 p.m. During the daytime (07:00 a.m.–6:00 p.m.), RH inside the barn remained elevated (≥60%). In general, RH was higher inside the barn than outside from 08:00 a.m. to 6:00 p.m., whereas the opposite pattern occurred between 6:15 p.m. and 7:45 a.m. Mean RH values were similar between seasons, averaging 78.6% in the cold season (range: 52.6–97.8%) and 80.91% in the hot season (range: 69.8–97.1%).

## 4. Discussion

The results demonstrated that characteristics of bedded-pack barn groups and seasonal climatic conditions significantly influenced bedding temperature, moisture, and physical–chemical properties. It is important to note that the monitoring period was designed to characterize spatial and diurnal bedding conditions within representative cold and hot seasons, rather than to describe long-term composting process dynamics. Differences among the evaluated CONV, ADAP, and PART systems indicate that management practices—particularly bedding turning and stocking density—play a central role in determining composting performance in systems with active composting (CBP).

The differences observed among bedded-pack systems can be mechanistically explained by the interaction between barn design and key composting processes. In CONV and ADAP systems, the combination of bedding turning and mechanical ventilation likely enhanced oxygen diffusion and moisture evaporation, creating favorable aerobic conditions for microbial activity and heat generation, which explains the higher temperatures observed. The temporary decrease in temperature immediately after turning, followed by a gradual increase, reflects heat loss due to material exposure and subsequent microbial reactivation as oxygen is reintroduced into deeper layers. In contrast, the absence of turning in PART systems resulted in higher bedding density and reduced porosity, limiting oxygen availability and favoring moisture retention, as evidenced by the higher moisture content and lower temperatures—indicating limited or absent composting activity in these systems. Additionally, differences in bedding depth and particle size distribution influenced pore structure, further regulating air movement and moisture dynamics within the pack. Together, these factors determine the physicochemical environment of the bedding and directly control composting performance in CBP systems, while constraining or preventing composting processes in BPB systems.

### 4.1. Bedded-Pack Temperature and Moisture

Bedding temperatures were higher during the hot season across all evaluated systems. In this study, daytime bedding temperatures ranged from approximately 20 to 50 °C. Mean bedding temperature was significantly higher in the ADAP system compared with CONV and PART, indicating a persistent group-level difference in the thermal conditions of the bedding. Despite spatial variability among sampling points within barns, the ADAP system consistently maintained higher temperatures throughout the experimental period, suggesting that these differences were associated with management and group characteristics rather than localized thermal effects.

It is suggested that the average bedding temperature should remain above 40 °C [1,3,12,29]. Some authors indicate that temperatures below 40 °C generally reflect reduced microbial activity and slower organic matter degradation, whereas temperatures above 55 °C are typically required for pathogen reduction [1,3,29]. The average bedding temperature reported in this study represents the mean of 20 sampling points within the bedded pack and may have been influenced by location-specific characteristics. Therefore, the observed temperatures do not necessarily indicate the absence of biological activity but suggest that composting likely occurred predominantly within the mesophilic range. Similar temperature ranges have been reported in compost bedded-pack studies conducted in North America, Europe, and Brazil [3,14,16,26,29,30].

Seasonal variation also influenced bedding temperature. During the cold season, lower air temperatures coincided with lower bedding temperatures across all evaluated systems, confirming the expected seasonal influence on pack thermal conditions, consistent with previous findings [3,14,17,26,29,31]. This relationship has been attributed to the greater thermal gradient between bedding and ambient air, which increases heat loss from the composting layer [3,14,29]. Colder weather increases the temperature gradient between ambient air and the bedded-pack barn, intensifying conductive and evaporative heat losses from the bedding surface and consequently reducing internal bedding temperatures [3]. Environmental conditions in southern Brazil, characterized by high relative humidity and moderate temperatures, may further limit moisture evaporation and bedding drying capacity, which can reduce microbial composting activity and heat generation within the pack [4,16]. Together, these factors contribute to lower heat retention and reduced composting performance in CBP systems during colder periods. In addition, providing greater resting space per cow during winter may reduce the moisture load per unit area, thereby decreasing the need for frequent bedding replacement or additional bedding supply [3]. Mechanical ventilation using fans can also increase air velocity and improve the drying rate of the bedding material [3].

Bedding turning strongly influenced bedding conditions. In the CONV and ADAP systems, bedding temperatures decreased immediately after turning events and subsequently increased, often reaching peak values approximately eight hours later. This pattern likely reflects temporary cooling caused by exposure of deeper layers during turning, followed by a gradual temperature increase associated with improved aeration and oxygen availability, which may enhance biological processes and heat generation [5,10,12,16,29].

In contrast, the PART system exhibited nearly constant temperatures throughout the day, reinforcing the importance of aeration in composting dynamics in CBP systems. In the absence of turning, oxygen availability may become limited, contributing to reduced heat generation and more stable thermal profiles, resulting in limited or absent composting activity. Although oxygen concentration was not directly measured, this study provides novel insights into hourly temperature patterns under field conditions.

Lower bedding temperatures were consistently observed in the PART group, which also showed higher bedding density and minimal daily thermal variation. The absence of turning likely restricts aeration and limits heat generation within the bedding. In addition to aeration, the selection of materials with adequate porosity is essential [9]. Consequently, the function of bedded-pack as a biologically active composting matrix is maintained in CBP systems but may be constrained or absent in BPB under conditions of limited aeration or increased compaction.

A significant group × season interaction was observed for moisture content. During the hot season, the PART system exhibited higher moisture content compared with CONV and ADAP. In contrast, during the cold season, all systems showed reduced drying capacity, likely due to regional climatic conditions, including lower temperatures, reduced solar radiation, and higher relative humidity. These factors likely limited moisture evaporation across all systems, regardless of structural differences.

Regardless of the system, bedding moisture remained within the range generally considered suitable for biological activity (approximately 40–60%) [12]. However, correlation analysis revealed a negative association between moisture and temperature, indicating that higher moisture levels were associated with lower bedding temperatures. Although within the recommended range, moisture levels approaching the upper limit may restrict aeration by reducing pore space, thereby limiting oxygen diffusion and potentially impairing heat generation and pathogen reduction.

The higher moisture content observed in the PART system is likely associated with limitations in moisture removal rather than increased water input. The lack of turning restricts aeration and prevents moisture redistribution within the bedding, while the absence of mechanical ventilation reduces airflow at the surface, limiting evaporative drying [32]. Furthermore, reduced heat generation further decreases evaporation potential, contributing to moisture accumulation within the bedding and conditions incompatible with active composting [32,33]. Areas with intense animal traffic—such as zones near water troughs or feed alleys—tended to present lower temperatures and higher moisture. High traffic may increase compaction and reduce pore space, limiting heat generation [14]. This interpretation is supported by the positive correlation observed between bedding moisture and density.

Barn structure also influenced composting dynamics. ADAP farms exhibited higher bedding temperatures, likely due to a combination of interacting factors rather than a single determinant. These systems generally presented greater bedding area per cow and were equipped with mechanical ventilation, which enhances airflow and promotes moisture removal [32]. Improved aeration and reduced moisture favor heat accumulation within the bedding [33]. In addition, structural characteristics such as roofing configuration may have contributed to reduced heat loss and more stable internal conditions. Together, these factors likely created more favorable conditions for heat generation compared with CONV and PART systems.

Within the ADAP group, Farms 4 and 6 were adapted from broiler sheds and featured lower ceiling heights (5.1 m and 3.0 m, respectively). The use of mechanical ventilation, combined with a larger bedding area per cow, may have contributed to the higher average bedding temperatures observed across both seasons.

The PART group maintained the lowest average bedding temperatures, with values ranging from 20 to 32 °C, indicating reduced heat generation and lower biological activity [21]. This study is among the first to evaluate composting dynamics in systems without bedding turning, reinforcing the importance of aeration for maintaining heat production within CBP systems. The observed thermal range in the PART group may also have been influenced by lower bedding depth, as these variables were moderately correlated (r = 0.39; Table 4). Additional factors such as stocking density, absence of mechanical ventilation, and structural limitations may have further contributed to moisture accumulation and reduced heat generation.

Finally, although variation in bedding temperature was observed among sampling points, no statistical analysis was performed to directly assess the relationship between climatic variables and bedding conditions. Therefore, any inference regarding the influence of air temperature and relative humidity on spatial variation should be interpreted with caution. Management practices, animal movement, and manure distribution likely contributed to the observed patterns. It should be noted that, although bedding turning frequency and stocking density are key factors influencing bedding dynamics, their individual effects were not explicitly evaluated in the present study, as these variables were intrinsically associated with the defined system typologies. Future studies under controlled experimental conditions are warranted to isolate and quantify the specific contribution of these management factors.

### 4.2. Physical Characteristics of the Bedding

Physical characteristics of bedding materials are key determinants of composting performance in composting bedded-pack systems. Properties such as APS distribution, porosity, moisture content, and bulk density directly influence the physical structure of the bedded pack and its ability to sustain microbial activity [9]. These attributes regulate important processes such as aeration, moisture retention, and microbial access to organic substrates within the bedding matrix.

APS distribution is particularly relevant because it affects both microbial colonization and airflow within the bedding material. Smaller particles increase the available surface area for microbial growth, which can enhance composting processes. However, excessively fine materials may promote compaction and reduce pore space, limiting oxygen availability and potentially inhibiting aerobic microbial activity [9]. Therefore, selecting bedding materials with an appropriate particle size distribution is essential for maintaining adequate composting conditions in bedded pack systems with active composting (CBP).

The predominance of intermediate particle sizes observed in the ADAP system may be related to routine bedding turning, and the presence of biologically active composting, which limits the accumulation of large particles (APS ≥ 9.50 mm) and promotes a more homogeneous bedding structure. Regular turning contributes to mechanical fragmentation and redistribution of bedding material, which can help maintain adequate porosity and microbial access to organic substrates.

On the other hand, the excessive accumulation of very fine particles may increase the risk of compaction, reducing oxygen availability and potentially limiting composting efficiency in CBP systems [33]. In this study, higher proportions of fine particles (<2 mm) were associated with higher bedding temperatures and lower moisture levels, suggesting that the CBP systems evaluated were able to manage the bedded pack in a way that maintained it adequately dry and in active composting.

Bedding density also reflected these dynamics. Bedding densities observed in this study were higher than values reported in previous Brazilian CBP studies [16,30]. Elevated bedding density is generally undesirable, as it limits oxygen availability [33]. The larger particle size observed in the PART system likely reflects excessive moisture content, which promoted the aggregation of particles that were not broken down due to the absence of turning, contributing to limited or absent composting activity. These combined factors contributed to the excessive bedding density (851 kg m^−3^).

Bedding pH values were consistently above the range typically considered optimal for composting processes (6.5–8.0). Elevated pH values are frequently reported in compost bedded-pack (CBP) systems and are commonly associated with the accumulation of nitrogen compounds resulting from manure and urine deposition [16,21].

The present study also identified a positive correlation between bedding pH and temperature, suggesting that higher microbial activity and organic matter degradation may contribute to increased alkalinity within the bedding, particularly in CBP systems.

### 4.3. Practical Implications

Overall, the results highlight that the efficiency of composting in bedded-pack barn (CBP) systems depends on the interaction between environmental conditions and bedding management practices.

As this experiment was conducted while preserving the specific management characteristics of each farm, the results support the potential implementation of these findings in other commercial composting bedded-pack barns. In practice, adapting CBP facilities to incorporate basic principles such as adequate ventilation, regular bedding turning, and maintenance of optimal moisture content can ensure good bedding quality and efficient composting performance. However, in the PART group, the absence of bedding turning remains a limiting factor for proper composting, representing a challenge for achieving desirable bedding conditions under this management system.

The results of this study provide several practical insights for the management of composting bedded-pack barns under humid subtropical conditions. From a farm-level perspective, the observed associations between bedding temperature, moisture content, particle size distribution, and density emphasize the relevance of bedding management practices for maintaining suitable bedding conditions. Systems that combined regular turning with sufficient ventilation capacity—such as the CONV and ADAP groups—showed more favorable thermal and moisture conditions, which are indicators of a biologically active and manageable bedding pack.

For farmers, monitoring bedding temperature and moisture can serve as simple, low-cost indicators to support daily management decisions. Temperatures persistently below the mesophilic range, combined with high moisture and increased bedding density, may signal inadequate aeration or excessive compaction, particularly in CBP systems, prompting adjustments in turning frequency, stocking density, or ventilation strategies.

From an environmental perspective, maintaining appropriate moisture levels and bedding structure is critical to reducing anaerobic conditions, and inefficient organic matter degradation. Improved moisture control through adequate aeration and bedding management enhances the environmental performance of composting bedded-pack system by favoring aerobic microbial processes and reducing the accumulation of excess moisture within the pack.

Economically, the findings suggest that management strategies promoting stable bedding conditions can contribute to longer bedding lifespan, reduced need for frequent material replacement, and lower labor and operational costs [3]. Additionally, better bedding quality may indirectly support animal welfare and health, potentially reducing expenditures related to mastitis, lameness, and hygiene-related issues [3].

Overall, the results emphasize that successful CPB performance under subtropical conditions depends not on individual design or management factors in isolation, but on their combined and consistent implementation. Farmers and technicians should therefore consider composting bedded-pack systems as integrated housing-management units, in which structural design, seasonal climate, and daily management practices jointly determine bedding quality, environmental impact, and economic sustainability.

## 5. Conclusions

The barn structure, bedding management, and seasonal conditions influenced composting dynamics in the evaluated compost-bedded pack barns. During the monitoring period, the ADAP system exhibited higher bedding temperatures and more favorable moisture conditions than the other systems, suggesting that structural characteristics and management practices may support microbial activity within the pack. However, these results should be interpreted with caution, as they reflect the combined effect of appropriate structural adjustments and effective management practices, rather than adaptation alone. In contrast, the PART system, classified as a non-composting bedded-pack system (BPB) showed lower and more stable bedding temperatures and higher moisture levels, indicating limited or absent composting activity under the evaluated conditions. These findings highlight that, in the absence of key management practices such as bedding turning and adequate aeration, bedded-pack systems may not function as biologically active composting systems. Seasonal climatic conditions also affected bedding characteristics, with the cold period generally associated with higher moisture and reduced drying capacity, which can further limit microbial activity, whereas systems without these practices operated under conditions incompatible with composting. This study reinforces the importance of distinguishing between composting (CBP) and non-composting bedded-pack systems (BPB), as these represent functionally different housing systems with distinct physical and biological dynamics.

## Figures and Tables

**Table 1:** Description of farms with bedded-pack barn systems contrasting composting functionality (CBP and BPB) in Santa Catarina State, Brazil.

Group	CONV			ADAP			PART		
	1	2	3	4	5	6	7	8	9
Bedding area (m^2^)	899	1505	1400	1176	1470	1200	236	220	480
Bedding depth (cm)	33.4	18.6	50.6	39.2	36.4	45.1	13.6	23.2	24.3
Bedding turning	Twice a day	Twice a day	Twice a day	Twice a day	Twice a day	Twice a day	Absent	Absent	Absent
Bedding material	Wood shavings	Wood shavings	Wood shavings	Wood shavings	Wood shavings	Wood shavings	Wood shavings	Wood shavings	Wood shavings
Length × width (m)	62.0 × 14.5	70.0 × 21.5	70.0 × 20.0	80.0 × 14.7	70.0 × 21.0	100.0 × 12.0	62.0 × 3.80	19.80 × 5.50	40.0 × 12.0
Ceiling Height (m)	5.7	6.5	4.4	5.1	6.0	3.0	2.4	3.2	4.8
Fans, (n and hp ^1^)	5.0 (2.5)	6.0 (2.0)	16.0 (2.0)	5.0 (2.0)	4.0 (1.5)	4.0 (2.0)	Absent	Absent	Absent
Drinker Length ^2^	14.3	19.5	7.3	9.1	24.4	6.1	9.6	4.5	6.1
Stocking Rate (m^2^/cow)	10.7	24.5	12.1	16.8	28.3	16.4	9.1	3.4	8.0
Number of Lactating Cows	83	61	123	70	52	73	26	32	60
Solar orientation ^3^	NS	NS	NS	EW	EW	NS	EW	NS	NS

^1^ hp: horsepower. ^2^ Length in centimeters of liners of drinkers per animal. ^3^ east–west—EW; north–south—NS. CONV: large conventional compost bedded-pack barns (CBP). ADAP: conventional or adapted compost bedded-pack barns. PART: non-composting bedded-pack barns (BPB) with partial use and access to grazing areas.

**Table 2:** Physical and chemical properties of bedded-pack barn systems with contrasting composting functionality (CBP and BPB) evaluated in Santa Catarina State, Brazil.

Variable	CONV	ADAP	PART	COLD	HOT	*p*–Value		
	Mean ± SD	Mean ± SD	Mean ± SD	Mean ± SD	Mean ± SD	Group	Season	Interaction
Temperature (°C)	37.39 ± 6.62 b	40.01 ± 4.51 a	26.18 ± 3.98 c	32.12 ± 7.55 b	36.55 ± 7.77 a	***	***	NS
Moisture content (%)	51.97 ± 10.14	47.75 ± 7.17	55.34 ± 7.28	54.62 ± 6.67	48.88 ± 10.01	***	***	***
pH	9.53 ± 0.27 b	9.75 ± 0.23 a	9.27 ± 0.48 c	9.39 ± 0.39 b	9.62 ± 0.37 a	***	***	NS
Density (kg m^−3^)	685.71 ± 258.19 b	702.70 ± 105.08 b	851.03 ± 186.92 a	748.06 ± 224.96	752.12 ± 193.12	***	NS	NS
Bedding depth (cm)	34.22 ± 17.63 b	39.28 ± 8.10 a	20.29 ± 6.46 c	31.31 ± 11.20	30.38 ± 17.32	***	NS	NS
APS (particle size distribution in %)								
≥9.50 mm	42.22 ± 23.78	45.62 ± 12.57	57.09 ± 15.88	48.53 ± 17.47	48.40 ± 21.34	***	NS	*
≥4.75 < 9.50 mm	14.81 ± 3.98 ab	16.60 ± 3.14 a	14.05 ± 4.42 b	14.83 ± 4.31	15.34 ± 3.71	***	NS	NS
≥2.00 < 4.75 mm	11.88 ± 4.18 ab	13.43 ± 2.46 a	10.44 ± 3.47 b	12.15 ± 3.51	11.46 ± 3.84	***	NS	NS
<2.00 mm	31.09 ± 25.71	24.35 ± 11.22	18.42 ± 13.13	24.49 ± 19.59	24.81 ± 18.12	***	NS	*

CONV: large conventional bedded-pack barn group. ADAP: conventional or adapted bedded-pack barn group. PART: non-composting bedded-pack barns (BPB) with partial use and access to grazing areas) and access to grazing areas. Means within the same row followed by different lowercase letters (a–c) differ significantly between groups or between seasons. NS = not significant (*p* > 0.05); * *p* ≤ 0.05; *** *p* ≤ 0.001. APS: average particle size, expressed as the percentage of bedding material within each particle size range (mm).

**Table 3:** Season × bedded-pack group interaction and simple effects for moisture, and particle size distribution of bedded-pack barns at 0.20 m depth.

Variable	Season	Group		
		CONV ^1^	ADAP ^2^	PART ^3^
Moisture (%)	Cold	57.03 aA	51.97 aA	54.86 aA
	Hot	46.91 bB	41.41 bB	55.83 aA
Average particle size ≥ 9.50 mm (%)	Cold	41.90 bA	51.65 aA	52.04 aA
	Hot	42.54 aA	36.57 bA	62.14 aA
Average particle size < 2.00 mm (%)	Cold	31.12 aA	18.92 aA	23.38 aA
	Hot	31.07 aA	32.43 aA	13.46 bA

^1^ CONV: large conventional bedded-pack barn group. ^2^ ADAP: conventional or adapted bedded-pack barn group. ^3^ PART: non-composting bedded-pack barns (BPB) with partial use and access to grazing areas. Different lowercase letters indicate significant differences among bedded-pack groups within the same season, whereas different uppercase letters indicate significant differences between seasons (cold vs. hot) within the same group (*p* ≤ 0.05).

**Table 4:** Pearson correlation matrix of physical and chemical variables of bedded-packs from nine farms evaluated during cold and hot seasons.

Variable	Temperature	Moisture	pH	Density	Depth	APS ≥ 9.50 mm	APS ≥ 4.75 < 9.50 mm	APS ≥ 2.00 < 4.75 mm	APS < 2.00 mm
Temperature	1	−0.41 *	0.53 *	−0.45 *	0.39 *	−0.49 *	0.13	0.34 *	0.41 *
Moisture		1	−0.15	0.27 *	0.21 *	0.52 *	0.15	−0.22 *	−0.52 *
pH			1	0.04	0.35 *	−0.02	0.30 *	0.24 *	−0.09
Density				1	0.01	0.67 *	0.03	−0.15	−0.67 *
Depth					1	0.18 *	0.19 *	0.09	−0.24 *
APS ≥ 9.50 mm						1	−0.01	−0.46 *	−0.93 *
APS ≥ 4.75 < 9.50 mm							1	0.64 *	−0.33 *
APS ≥ 2.00 < 4.75 mm								1	0.14
APS < 2.00 mm									1

Pearson correlation coefficients (r) among physical and chemical variables of bedded-packs from nine farms evaluated during cold and hot seasons. Values represent correlation coefficients, and asterisks (*) indicate significant correlations (*p* ≤ 0.05). Only significant correlations are highlighted. APS: average particle size, expressed as the percentage of bedding material within each particle size range (mm). Note: Cell shading intensity indicates the magnitude of Pearson’s correlation coefficients. Darker shades represent stronger correlations, whereas lighter shades represent weaker correlations. Positive and negative correlations are represented by the numerical values shown in the cells.

## Data Availability

The data presented in this study are available from the corresponding author upon reasonable request. Data are not publicly available due to confidentiality agreements with participating farms.

## References

[1] Damasceno F.A., Oliveira C.E.A., Ferraz G.A.S., Nascimento J.A.C., Barbari M., Ferraz P.F.P. (2019). Spatial distribution of thermal variables, acoustics and lighting in compost dairy barn with climate control system. Agron. Res..

[2] Vieira F.M.C., Soares A.A., Herbut P., Vismara E.S., Godyń D., dos Santos A.C.Z., Lambertes T.S., Caetano W.F. (2021). Spatio-thermal variability and behaviour as bio-thermal indicators of heat stress in dairy cows in a compost barn: A case study. Animals.

[3] Black R.A., Taraba J.L., Day G.B., Damasceno F.A., Bewley J.M. (2013). Compost bedded pack dairy barn management, performance, and producer satisfaction. J. Dairy Sci..

[4] Pilatti J.A., Vieira F.M.C., Rankrape F., Vismara E.S. (2019). Diurnal behaviors and herd characteristics of dairy cows housed in a compost-bedded pack barn system under hot and humid conditions. Animal.

[5] Eberl D.T., Smith M.J., Megram O.J., Mayhew M.M., Willoughby D., White S.J., Wilson P.B. (2024). innovative bedding materials for compost bedded pack barns: Enhancing dairy cow welfare and sustainable dairy farming. Environ. Dev. Sustain..

[6] Janni K.A., Endres M.I., Reneau J.K., Schoper W.W. (2007). Compost dairy barn layout and management recommendations. Appl. Eng. Agric..

[7] Andrade R.R., Tinôco I.F.F., Damasceno F.A., Oliveira C.E.A., Concha M.S., Zacaroni O.F., Bambi G., Barbari M. (2024). Understanding compost-bedded pack barn systems in regions with a tropical climate: A review of the current state of the art. Animals.

[8] Damasceno F.A., Day G.B., Taraba J.L., Oliveira C., Andrade R.R., Frigeri K., Vieira F., Barbari M., Bambi G. (2022). Compost dairy barn layout and management recommendations in Kentucky: A descriptive study. Animals.

[9] Ferraz P.F.P., Ferraz G.A.S., Leso L., Klopčič M., Barbari M., Rossi G. (2020). Properties of conventional and alternative bedding materials for dairy cattle. J. Dairy Sci..

[10] Black R.A., Taraba J.L., Day G.B., Damasceno F.A., Newman M.C., Akers K.A., Wood C.L., McQuerry K.J., Bewley J.M. (2014). The relationship between compost bedded pack performance, management, and bacterial counts. J. Dairy Sci..

[11] Albino R.L., Taraba J.L., Marcondes M.I., Eckelkamp E.A., Bewley J.M. (2018). Comparison of bacterial populations in bedding material, on teat ends, and in milk of cows housed in compost bedded pack barns. Anim. Prod. Sci..

[12] Bewley J.M., Taraba J.L., McFarland D., Garrett P., Graves R., Holmes B., Kammel D., Porter J., Tyson J., Weeks S. (2013). Guidelines for Managing Compost Bedded-Pack Barns.

[13] Barberg A.E., Endres M.I., Janni K.A. (2007). Compost dairy barns in Minnesota: A descriptive study. Appl. Eng. Agric..

[14] Leso L., Uberti M., Morshed W., Barbari M. (2013). A survey on Italian compost dairy barns. J. Agric. Eng..

[15] Biasato I., D’Angelo A., Bertone I., Odore R., Bellino C. (2019). Compost bedded-pack barn as an alternative housing system for dairy cattle in Italy: Effects on animal health and welfare and milk and milk product quality. Ital. J. Anim. Sci..

[16] Radavelli W.M., Danieli B., Zotti M.L.A.N., Gomes F.J., Endres M.I., Schogor A.L.B. (2020). Compost barns in Brazilian Subtropical region (Part 2): Classification through multivariate analysis. Res. Soc. Dev..

[17] Oliveira C.E.A., Tinôco I.F.F., Oliveira V.C., Rodrigues P.H.M., Silva L.F., Damasceno F.A., Andrade R.R., Sousa F.C., Barbari M., Bambi G. (2023). Spatial distribution of bedding attributes in an open compost-bedded pack barn system with positive pressure ventilation in Brazilian winter conditions. Animals.

[18] Alvares C.A., Stape J.L., Sentelhas P.C., Gonçalves J.L.M., Sparovek G. (2013). Köppen’s climate classification map for Brazil. Meteorol. Z..

[19] Oliveira C.E.A., Tinôco I.d.F.F., Sousa F.C.d., Baêta F.d.C., Vieira F.M.C., Barbari M. (2024). Health and thermal comfort of dairy cattle in compost-bedded pack barns and other types of housing: A comparative systematic review. AgriEngineering.

[20] (2007). Cubes, Pellets and Crumbles—Definitions and Methods for Determining Density, Durability and Moisture Content.

[21] Silva D.J., Queiroz A.C. (2002). Análise de Alimentos: Métodos Químicos e Biológicos.

[22] Damasceno F.A. (2012). Compost Bedded Pack Barns System and Computational Simulation of Airflow Through Naturally Ventilated Reduced Model. Ph.D. Thesis.

[23] Maia G.D.N. (2010). Ammonia Biofiltration and Nitrous Oxide Generation as Affected by Media Moisture Content. Ph.D. Dissertation.

[24] Autodesk (2015). AutoCAD.

[25] Jakob A.A.E., Young A.F. (2006). O uso de métodos de interpolação espacial de dados nas análises sociodemográficas. Proceedings of the XV Encontro Nacional de Estudos Populacionais.

[26] Andrade R.R., Tinôco I.F.F., Damasceno F.A., Ferraz G.A.S., Freitas L.C.S.R., Ferreira C.F.S., Barbari M., Baptista F.J.F., Coelho D.J.R. (2022). Spatial distribution of bed variables, animal welfare indicators, and milk production in a closed compost-bedded pack barn with a negative tunnel ventilation system. J. Therm. Biol..

[27] QGIS Development Team (2019). QGIS Geographic Information System.

[28] R Core Team (2017). R: A Language and Environment for Statistical Computing.

[29] Eckelkamp E.A., Taraba J.L., Akers K.A., Harmon R.J., Bewley J.M. (2016). Understanding compost bedded pack barns: Interactions among environmental factors, bedding characteristics, and udder health. Livest. Sci..

[30] Fávero S., Portilho F.V.R., Oliveira A.C.R., Langoni H., Pantoja J.C.F. (2015). Factors associated with mastitis epidemiologic indexes, animal hygiene, and bulk milk bacterial concentrations in dairy herds housed on compost bedding. Livest. Sci..

[31] Llonch L., Castillejos L., Mainau E., Manteca X., Ferret A. (2020). Effect of forest biomass as bedding material on compost-bedded pack performance, microbial content, and behavior of nonlactating dairy cows. J. Dairy Sci..

[32] Leso L., Ferraz P.F.P., Ferraz G.A.S., Rossi G., Barbari M. (2021). Factors Affecting Evaporation of water from cattle bedding materials. Biosyst. Eng..

[33] Ferraz P.F.P., Araújo e Silva Ferraz G., Leso L., Klopčič M., Rossi G., Barbari M. (2020). Evaluation of the physical properties of bedding materials for dairy cattle using fuzzy clustering analysis. Animals.

